# 肺癌PDTX模型的研究进展

**DOI:** 10.3779/j.issn.1009-3419.2017.10.09

**Published:** 2017-10-20

**Authors:** 保东 秦, 晓栋 焦, 凌燕 原, 珂 柳, 远胜 臧

**Affiliations:** 200003 上海，上海长征医院肿瘤科 Department of Medical Oncology, Shanghai Changzheng Hospital, Shanghai 200003, China

**Keywords:** 肺肿瘤, PDTX模型, 精准治疗, Lung neoplasms, Patient derived tumor xenograf, Precision therapy

## Abstract

当前随着肿瘤分子生物学及基因组学的发展，人们已经认识到同一瘤种在不同个体间其生物学特征、分子分型以及对药物干预的反应性都存在巨大的异质性，这种个体化差异是导致肿瘤治疗过程中同病同治而不同效的重要原因，因此为了实现真正的肿瘤个体化精准治疗，肿瘤研究领域提出了一个新的概念即人源肿瘤组织异种移植模型（patient derived tumor xenograft, PDTX）；该模型可以真实地反映患者肿瘤组织的生物学特性以及药物疗效，是研究个体化治疗、药物耐药以及新药研发的重要手段，已被运用包括肺癌在内多个瘤种的临床诊治过程中。本文就当前肺癌PDTX模型的研究进展进行综述。

## Abstract (en)

With the development of tumor molecular biology and genomics, it has been recognized that there are great heterogeneity in the biological characteristics, molecular typing and reactivity of the same tumor species among different individuals. In order to achieve true tumor individualized and precise therapy, a new concept of human tumor tissue xenograft model (patient derived tumor xenograft, PDTX) is proposed. The previous study has revealed that PDTX model can truly reflect the biological characteristics of tumor tissue and drug efficacy. And PDTX model could be used to select individual chemotherapy regime, evaluate drug resistance and explore efficacy and safety of new drug. PDTX model has been used in clinical practice of several type of cancer including lung cancer. In this paper, the current research progress of lung cancer PDTX is reviewed.

当前随着肿瘤分子生物学及基因组学的发展，人们已经认识到同一瘤种在不同个体间其生物学特征、分子分型以及对药物干预的反应性都存在巨大的异质性，这种个体化差异是导致肿瘤治疗过程中同病同治而不同效的重要原因，因此为了实现真正的肿瘤个体化精准治疗，肿瘤研究领域提出了一个新的概念，即人源肿瘤组织异种移植模型（patient derived tumor xenograft, PDTX），该模型可以真实地反映患者肿瘤组织的生物学特性以及药物疗效，是研究个体化治疗、药物耐药以及新药研发的重要手段，已被运用包括肺癌在内多个瘤种的临床诊治过程中。本文就当前肺癌PDTX模型的研究进展进行综述如下。

## 1 PDTX模型简介

1990年开始，肿瘤学界已用NCI-60系列肿瘤细胞筛选出了10万余种化合物用于肿瘤发病分子机制的探讨以及相关药物研发，但由于该类细胞长期传代逐渐适应了与原生环境完全不同的培养皿环境，肿瘤细胞基因组学背景与生物学行为已与原代细胞大相径庭，不能有效地、真实地反映肿瘤状态，因此2016年美国癌症研究所已下架NCI-60系列的60株肿瘤细胞，开始转向肿瘤移植动物模型^[1]^。由于基因编辑较易、传代周期较短以及研究成本较低等优点，小鼠模型一直以来是肿瘤研究中最被广泛使用的系统之一^[2]^。传统的人源肿瘤细胞异种移植是将人类肿瘤细胞在体外筛选，经过传代培养建立稳定的细胞株，然后移植至裸鼠中构建模型，半个世纪以来，这种模型的建立已被广泛应用于肿瘤的基础研究与临床探索中。但由于连续传代的肿瘤细胞株适应了培养皿的环境，缺乏肿瘤微环境如成纤维细胞、免疫细胞、细胞外基质、肿瘤微环境因子等，这些细胞株种植到免疫缺陷小鼠后形成的肿瘤是同质性的，丢失了原代肿瘤的特性，不能客观地、真实地反映原代肿瘤的情况，因此人源肿瘤组织异种移植模型即PDTX模型应运而生。通过外科手术或活检获得肿瘤患者的肿瘤组织，通过皮下移植、肾包膜下移植或原位移植等方式移植于免疫缺陷小鼠中，随后进行小鼠数量扩增，给予不同的药物或药物组合方案干预，进行药效学评价，再根据药效学评估结果指导肿瘤病人临床治疗策略选择，实现从基础实验到临床应用的转化^[3]^。

## 2 影响肺癌PDTX建模的因素

以往研究报道不同瘤种PDTX模型的构建成功率存在差异，结肠癌的建模成功率约为43%，急性淋巴细胞白血病的建模成功率最高达64%，乳腺癌的建模成功率较低仅为9%，肺癌的建模成功率约为18%-23%^[4-7]^。在肺癌PDTX模型构建过程中，诸多因素影响着肿瘤模型成功与否，其中最重要的因素为肿瘤组织的质量与活力，其他因素包括肿瘤组织大小、植入时间、辅助化疗的间隔时间、裸鼠的免疫缺陷程度等，而除了这些宏观因素之外，近来研究还发现肺癌PDTX建模成功与肿瘤的细胞类型、恶性程度等密切相关；如研究发现肺腺癌患者建模成功率要显著低于鳞癌与神经内分泌肿瘤，中分化与低分化的患者成瘤率要显著高于高分化^[8, 9]^，同样有研究表明年轻患者肺癌PDTX建模成功率高于年老患者^[10]^，PD-L1高表达患者成功率高于低表达患者^[11]^。Stewart等^[10]^对比分析建模成功与未成功的肺癌组织基因表达情况，发现了163个异常表达基因，且这些基因功能主要富集于细胞周期/有丝分裂、细胞增殖等信号通路。除此之外，肿瘤组织的提取方式同样影响着建模成功率，有报道认为在所有瘤种中，手术标本建模成功率高达27.3%-70%，细针穿刺活检成功率仅为0%-36.4%^[12, 13]^。而在肺癌中，有研究表明通过支气管镜引导下经支气管针吸活检术（endobronchial ultrasound-guided transbronchial needle aspiration, EBUS-TBNA）获得肺癌转移淋巴结组织，PDTX建模成功率为42.1%^[14]^，而另一项针对小细胞肺癌（small cell lung cancer, SCLC）患者PDTX模型构建的研究发现EBUS-TBNA获取的肿瘤组织建模成功率高达83%，但由于样本数量的有限性，这些数据仍需要进一步证实^[15]^。

除了使用肺癌的原发灶构建PDTX模型外，Lee等^[16]^探讨了肺癌脑转移灶构建PDTX模型，发现非小细胞肺癌（non-small cell lung cancer, NSCLC）患者脑转移灶组织构建PDTX模型成功率显著高于肿瘤原发灶（74% *vs* 23%），且肿瘤组织原发灶PDTX成瘤率与组织特征、肿瘤临床侵袭能力以及基因突变相关，而脑转移灶PDTX成瘤率与患者临床特征、分子分型均无关。

## 3 肺癌PDTX模型与原代肿瘤生物学特征的一致性

许多研究评估了PDTX移植肿瘤与原代肿瘤间生物学特征的一致性，研究结果显示不同瘤种的PDTX移植肿瘤与原代肿瘤组织以及不同代数的PDTX移植肿瘤间在病理特征、基因表达以及驱动基因突变等方面均高度一致^[6, 17]^。在NSCLC中，Merk等^[9]^研究者证实PDTX移植肿瘤在组织病理类型、细胞表面分子表达如E-钙粘蛋白、EpCAM以及Ki67等均与原代肿瘤组织一致，同样在SCLC中，PDTX模型也被证实可以有效复制原代肿瘤组织的组织学类型、病理特征等^[18]^。

除了病理类型，研究还发现肺癌PDTX模型肿瘤与原代肿瘤间在基因突变方面也高度一致。Hao等对23例肺癌PDTX进行了基因突变分析，发现在原代肿瘤组织315个基因突变中，有包括*TP53*、*KRAS*、*PI3KCA*、*ALK*、*STK11*、*EGFR*在内的293个基因突变可在相应的PDTX模型中发现，二者符合度达93%，且证实F1代肿瘤组织与F3代肿瘤组织间基因突变同样呈高度一致，说明在小鼠传代过程中肿瘤组织基因突变信息是高度保守^[7, 8]^。随后，Wang等^[19]^研究者对比分析了36例NSCLC患者的PDTX模型，发现与肿瘤细胞系相比，PDTX模型的体细胞突变、基因拷贝数、表达谱、DNA甲基化、蛋白组学特征与原代肿瘤细胞有着更高的相似度。此外，上述两项研究均发现PDTX模型中存在原代肿瘤组织未检出的基因突变或低频突变频率升高的现象，对于该现象当前观点认为可能与PDTX模型中相应的亚克隆肿瘤细胞选择性生长所致。

除此之外，当前还有研究探讨了肺癌PDTX模型是否能够真实反映原代肿瘤组织的代谢特征，Valtorta构建了9例肺癌PDTX模型，通过PET-CT技术检测PDTX移植肿瘤的SUVmax，结果显示与原代肿瘤一致，PDTX移植肿瘤在^18^F-FDG摄取率、代谢率等方面与原代肿瘤高度相似，从而说明PDTX模型同样可以反映原代肿瘤的葡萄糖代谢功能^[20]^。

## 4 PDTX模型能够预测肺癌患者的药物疗效

Stewart等^[10]^纳入了33例*EGFR*突变的肺腺癌患者，通过外科手术标本构建了PDTX模型，并给予一代与二代TKI药物治疗，研究结果显示6例患者PDTX模型构建成功，而此6例患者对包括厄洛替尼在内的一代TKI的治疗有效率与PDTX模型的反应率一致。同样，Zhang等^[21]^构建了*EGFR*突变的NSCLC患者的PDTX模型并证实吉非替尼在PDTX模型中的抗肿瘤效果与临床患者相似；此外，Li等^[22]^比较了PDTX模型与分子信号图间在预测EGFR靶向药物在肺癌中的疗效选择靶向治疗优势人群间的区别，结果显示二者之间在预测西妥昔单抗疗效的一致率为84%，预测厄洛替尼疗效的一致性约为80%，因此认为PDTX模型方法可用于临床筛选靶向治疗优势人群。除了*EGFR*突变外，研究者还使用PDTX模型成功构建了FGFR1扩增的NSCLC患者PDTX模型，并证实FGFR1抑制剂（AZD4547）在NSCLC鳞癌患者中可以有效的抑制肿瘤生长且其抗肿瘤效果与患者的FGFR1 FISH评分及蛋白表达水平呈正相关性^[23, 24]^。而在SCLC患者中，研究者在一项二期临床试验中发现PDTX模型能够真实反映三氧化二砷在复发型SCLC患者中的临床治疗结局^[25]^。

除了预测相关药物疗效外，研究还发现PDTX模型可以预测肺癌药物耐药情况。如Merk等^[26]^证实PDTX模型可用于临床中治疗相关标志物的研究以及药物耐药动态监测，Nakagawa等^[27]^通过PDTX模型证实了ATP7B基因与铂类药物耐药密切相关。

## 5 PDTX模型能够预测肺癌患者临床预后

除了在肿瘤患者治疗方面的应用，有研究还发现PDTX成瘤率可以有效预测肿瘤患者的预后情况。研究分析结直肠癌患者的PDTX成瘤性与肿瘤恶性程度、预后的相关性时发现TNM分期晚、肿瘤细胞分化差的患者中PDTX成瘤性率升高，成瘤率与肿瘤的恶性程度、差的预后密切相关，证实PDTX成瘤性可用于判断结直肠癌临床预后^[28]^。DeRose等^[6]^的研究也证实无法成瘤或瘤体不生长的PDTX模型对应的乳腺癌患者具有更长的生存时间。在肺癌中，有研究报道显示PDTX成瘤情况同样可以有效预测肺癌患者的预后，PDTX成瘤阳性的*EGFR*突变肺腺癌患者的无病生存时间、总生存时间均短于成瘤阴性的患者，疾病进展风险与死亡风险分别增加4.88倍与8.44倍^[10]^；Wang等^[19]^研究同样证实NSCLC患者的PDTX成瘤性与患者的总生存时间密切相关，且PDTX成瘤阳性是NSCLC的一个独立的预后不良因素。

## 6 PDTX模型的不足之处

与传统的人源性肿瘤细胞异种移植相比，PDTX模型具有更高的肿瘤空间异质性、更多样的分子亚型、更丰富的肿瘤微环境，能够真实反映肿瘤的生物学特征。但是在看到优点的同时，我们应该注意到PDTX同样存在些许不足之处：①肿瘤异质性问题，PDTX模型仅仅需要2 mm^3^-3 mm^3^的肿瘤组织，由于肺癌肿瘤患者体内肿瘤组织存在显著的空间异质性，显然2 mm^3^-3 mm^3^的肿瘤组织无法完全代表原代肿瘤的所有生物学特性^[29]^；②尽管PDTX模型肿瘤组织中富含肿瘤微环境，但是在成瘤过程中人源细胞如成纤维细胞、免疫细胞、基质细胞、血管内皮细胞等将被相应的鼠源细胞所替代，这样的肿瘤微环境能否还原原代肿瘤微环境的特征同样值得进一步研究证实；③在PDTX模型中我们无法完全做到原位移植，因此小鼠中移植部位的不同可能会影响PDTX模型的真实性，肺癌患者肿瘤组织移植于小鼠皮下，无法真实还原肿瘤的组织环境特征、血供水平等；④由于PDTX模型所用载体为免疫缺陷小鼠，无法验证免疫治疗如PD-1/L1抗体的有效性等；此外，PDTX模型的构建周期较长、费用较高以及技术难度等均限制了其在临床应用与开展范围。

## 7 展望

综上所述，以往的研究说明PDTX模型在肿瘤药效学评价以及肿瘤临床预后判断等方面都有着重要的作用，已成为肿瘤研究中的一个热点。在Clinicaltrail数据库（https://www.clinicaltrials.gov）中检索当前共有20余项应用PDTX模型的临床试验得以注册，涉及到肺癌、头颈部肿瘤、乳腺癌、软组织肉瘤、胃肠道肿瘤等，主要的研究目的包括验证PDTX模型肿瘤与原代肿瘤组织病理学特征、基因特征的一致性，探讨某些新药或药物组合的疗效以及探寻药物疗效或药物耐药的相关生物学标志物等。我们可以看到这些研究紧扣当前肿瘤研究热点，集中于肿瘤领域有待解决的热点问题，随着这些研究项目的实施、研究结果的呈现以及PDTX模型技术的完善与提升，不久将来，PDTX模型将使肿瘤患者进入真正的私人定制时代。而为了解决肿瘤异质性问题，如上所述有研究者采用EBUS-TBNA技术能够在超声引导下穿刺提取高纯度的肺癌肿瘤组织，提高PDTX模型成功概率并最大限度降低肿瘤异质性对PDTX模型的影响，但这类研究仅限于小样本临床探索中，将来仍需要大样本研究进一步证实。同时，对于PDTX模型的原位移植研究当前仅限于肝癌、胰腺癌、前列腺癌及乳腺癌等瘤种中个案报道，对于肺癌PDTX模型的原位移植当前少有研究涉及，考虑肺癌原位移植为体内深部组织，结合基础研究中肿瘤细胞成瘤模型经验，支气管内注射或胸腔内原位注射法是否可成为肺癌PDTX模型构建的重要方法值得进一步研究证实。
